# Recombinant expression and antigenicity of two peptide families of neurotoxins from *Androctonus* sp.

**DOI:** 10.1590/1678-9199-JVATITD-2022-0026

**Published:** 2022-12-19

**Authors:** Samuel Cardoso-Arenas, Herlinda Clement, Iván Arenas, Felipe Olvera, Fernando Zamudio, Figen Caliskan, Ligia Luz Corrales-García, Gerardo Corzo

**Affiliations:** 1 Department of Molecular Medicine and Bioprocesses, Institute of Biotechnology, National Autonomous University of Mexico (UNAM), Cuernavaca, Morelos, Mexico.; 2 Department of Biology, Faculty of Science and Letters, Eskisehir Osmangazi University, Eskisehir, Turkey.; 3 Department of Food Sciences, School of Pharmaceutical and Food Sciences, University of Antioquia (UdeA), Medellín, Colombia.

**Keywords:** Androctonus, Antibodies, Protein expression, Scorpion venom

## Abstract

**Background:**

Scorpion neurotoxins such as those that modify the mammalian voltage-gated sodium ion channels (Nav) are the main responsible for scorpion envenomation. Their neutralization is crucial in the production of antivenoms against scorpion stings.

**Methods:**

In the present study, two *in silico* designed genes - one that codes for a native neurotoxin from the venom of the Anatolian scorpion *Androctonus crassicauda,* named Acra 4 - and another non-native toxin - named consensus scorpion toxin (SccTx) obtained from the alignment of the primary structures of the most toxic neurotoxins from the Middle Eastern and North African scorpions - were recombinantly expressed in *E. coli* Origami.

**Results:**

Following bacterial expression, the two expressed neurotoxins, hereafter named HisrAcra4 and HisrSccTx, were obtained from inclusion bodies. Both recombinant neurotoxins were obtained in multiple Cys-Cys isoforms. After refolding, the active protein fractions were identified with molecular masses of 8,947.6 and 9,989.1 Da for HisrAcra4 and HisrSccTx, respectively, which agreed with their expected theoretical masses. HisrAcra4 and HisrSccTx were used as antigens to immunize two groups of rabbits, to produce either anti-HisrAcra4 or anti-HisrSccTx serum antibodies, which in turn could recognize and neutralize neurotoxins from venoms of scorpion species from the Middle East and North Africa. The antibodies obtained from rabbits neutralized the 3LD_50_ of *Androctonus australis, Leiurus quinquestriatus hebraeus* and *Buthus occitanus* venoms, but they did not neutralize *A. crassicauda* and *A. mauritanicus* venoms. In addition, the anti-HisrAcra4 antibodies did not neutralize any of the five scorpion venoms tested. However, an antibody blend of anti-HisrAcra4 and anti-HisrSccTx was able to neutralize *A. crassicauda* and *A. mauritanicus* venoms.

**Conclusions:**

Two recombinant Nav neurotoxins, from different peptide families, were used as antigens to generate IgGs for neutralizing scorpion venoms of species from the Middle East and North Africa.

## Background

Scorpion venoms contain neurotoxins that act directly, or in synergism with other molecules from the same venom, on mammalian or insect cell receptors that cause envenomation symptoms, paralysis, or death [1-3]. There are more than 2,000 scorpion species known in the world, but the venom of only a few species is dangerous to humans [4]. Some of the scorpions that cause severe envenoming belong to the Buthidae family, which includes mainly scorpions from the genera *Leiurus* in the Middle East, and *Androctonus* and *Buthus* in North Africa. Their venoms exert inhibitory actions of two types of NTxs, namely Na^+^ channel toxins (NavTx) and K^+^ channel toxins (KvTx) [5], which are in relative abundance in the venoms of Middle Eastern and North African buthid scorpions. These two types of neurotoxins affect the voltage-gated Na^+^- and K^+^- ion channels, thus interfering with the generation and propagation of action potentials in neurons and also causing second metabolic events which are potentially lethal for human beings [6, 7].

The number of scorpion stings in this vast geographic area of the Middle East and North Africa has been estimated to be around 146,000 and 350,000, respectively, which, together, make up more than 40% of the global scorpion stings. Compared to other regions of the world, the mortality rate in the Middle East and North Africa are the highest. Some examples of countries with severe problems of scorpionism are Turkey, Algeria, Egypt, Morocco, and Tunisia among others [8]. Therefore, alternatives for producing antivenoms could improve the offer for public and private clinics to treat scorpion stings. 

Commercial antidotes or antivenoms against scorpion stings contain antibodies that recognize those neurotoxins and neutralize them. Antivenoms are produced basically by immunization of animals, mostly horses, with the complete venom “milked” from scorpions, which may contain toxic or non-toxic constituents. One of the drawbacks of the process of animal immunization is that, in certain cases, the whole venom used as immunogen is detrimental to the animals [9]. Another one is that the non-toxic constituents affect the antibody response, and “dilute” the number of the neutralizing antibodies against important toxic constituents. Therefore, the use of recombinant antigens that generate neutralizing antibodies against main neurotoxins could be an alternative for improving antivenom production.

Some representative species from the Middle East and North Africa scorpions are *Androctonus crassicauda*, A. *australis, A. mauritanicus, Leiurus quinquestriatus hebraeus,* and *Buthus occitanus*, whose venoms contain highly toxic neurotoxins. These scorpion venoms contain at least two families of neurotoxins that affect mammalian cell receptors and cause severe envenomation symptoms [10]. The two types of proteins differ slightly in their primary structures and belong to two distinct structural and immunological groups of neurotoxins. The mammalian neurotoxins Acra4, AaH1, and AaH3 represent one group, and the neurotoxins such AaH2, AmmV, and AmmVIII represent another group (see Table 1A and 1B). 

**Table 1.** Amino acid sequences, lethal dose, and identity of Acra4, SccTx and selected neurotoxins of scorpion venoms from North Africa and the Middle East.

A				
Neurotoxin	Amino acid sequence^1^	LD_50_ ng/mouse	Identity to Acra4 (%)	Identity to SccTx (%)
Acra4	VRDGYIVDDKNC VYH C IPP-- CDGLCKKNGGKSGSC SFLVPSGLACWCKALPDNVPIKDPSYKCHKR	50^a^	100	53.8
AmmV	LKDGYIIDDLNCTFFCGRNAYCDDECKKKGGESGYCQWASPYGNACWCYKLPDRVSIKEKG-RCN--	3.5^a^	51.6	78.1
Lqq5	LKDGYIVDDKNCTFFCGRNAYCNDECKKKGGESGYCQWASPYGNACWCYKLPDRVSIKEKG-RCN--	2.5^a^	53.2	82.8
AmmVIII	LKDGYIVNDINCTYFCGRNAYCNELCIKLKGESGYCQWASPYGNSCYCYKLPDHVRTKGPG-RCNDR	225^a^	46.8	89.4
Lqh2	IKDGYIVDDVNCTYFCGRNAYCNEECTKLKGESGYCQWASPYGNACYCYKLPDHVRTKGPG-RCR--	1.9^a^	48.3	92.2
Bot3	VKDGYIVDDRNCTYFCGRNAYCNEECTKLKGESGYCQWASPYGNACYCYKVPDHVRTKGPG-RCN--	25^a^	46.8	92.2
AaH2	VKDGYIVDDVNCTYFCGRNAYCNEECTKLKGESGYCQWASPYGNACYCYKLPDHVRTKGPG-RCH--	0.5^a^-220^b^	51.6	93.7
Consensus	+KDGYIVDD+NCTYFCGRNAYCNEEC+KLKGESGYCQWASPYG NACYCYKLPDHVRTKGPGYRCN+R	ND	-	-
SccTx	LKDGYIVDDKNCTYFCGRNAYCNEECKKLKGESGYCQWASPYG NACYCYKLPDHVRTKGPGYRCNKR	ND	53.8	100
**B**				
Acra4	VRDGYIVDDKNCVYHCIPP--CDGLCKKNGGKSGSCSFLVPSGLACWCKALPDNVPIKDPSYKCHKR	50^a^	100	53.8
AaH3	VRDGYIVDSKNCVYHCVPP--CDGLCKKNGAKSGSCGFLIPSGLACWCVALPDNVPIKDPSYKCHSR	7^a^	89.2	49.2
AaH1	KRDGYIVYPNNCVYHCVPP--CDGLCKKNGGSSGSCSFLVPSGLACWCKDLPDNVPIKDTSRKCTR	10^a^	82.8	43.7
AmmIII	GRDGYIVDTKNCVYHCYPP--CDGLCKKNQAKSGSCGFLYPSGLACWCVALPENVPIKDPNDDCHK	7^a^	81.2	46.9
Bot9	VRDGYIVYPNNCVYHCGLNPYCNDLC C TKWGNACYCYALPEKVPIKDPSYKCHS	2^a^	65.6	54.5
LqhVI	VRDGYIAQPENCVYHCIPD--CDTLCKDNGGTGGHCGFKLGHGIACWCNALPDNVGIIVDGVKCHK	34^a^-96.5^b^	64.1	39.1
SccTx	LKDGYIVDDKNCTYFCGRNAYCNEECKKLKGESGYCQWASPYG NACYCYKLPDHVRTKGPGYRCNKR	ND	53.8	100

^1^ Acra4 (M1JBC0.1) from *A. crassicauda;* AaH1 (P01479.3), AaH2 (P01484.3) and AaH3 (P01480.3) from *A. australis hector;* AmmIII (P0C910.1), AmmV (P01482.1) and AmmVIII (Q7YXD3.1) from *A. m. mauritanicus;* Lqq5 (P01481.1) from *L. quinquestriatus quinquestriatus;* Lqh2 (P59355.1) and Lqh6 (P59356.1) from *L. quinquestriatus hebraeus;* Bot3 (P01485.2) and Bot9 (P04099.2) from *B. occitanus tunetanus.*

^a^ Intracerebroventricular (icv). ^b^Subcutaneous (sc). Acra4 - icv [11]; AaH2 - icv and sc [12]; AmmV - icv [13]; Lqq5 - icv [14]; AmmVIII - icv [12]; Lqh2 - icv [15]; Bot3 - icv [16]; Aah3 - icv [17]; Aah1 - icv [18]; Amm III - icv [13]; Bot9 - icv [19]; LqhVI - icv and sc [20].

The toxins Acra4 and SccTx were used as models to raise antibodies in rabbits to explore the immunogenicity of these neurotoxins towards scorpion venoms. Several communications have reported recombinantly expressed neurotoxins from venoms of North Africa and the Middle East scorpion species to produce antivenoms (see Table 2). However, the present study addresses complement information that usually is not discussed in other reports, such as the refolding steps of expressed neurotoxins, the use of a blend of antibodies to tackle differences in neurotoxin group content in such scorpion venoms, and the use of these antibodies to neutralize other scorpion venoms from the Middle East and North Africa species. 

**Table 2.** Expressed recombinant neurotoxins obtained from scorpion venoms from North Africa and the Middle East.

Recombinant or native antigen	Scorpion origin	Source of antigen	Immunization	Target	Neutralization LD_50_ /mL plasma	Other scorpion venoms neutralized	References
Bot XIV fused to protein A from *S.* *aureus* (soluble)	*B. occitanus tunetanus*	*E. coli*	Mice	BotG-50 fraction	20	ND	[21]
Bot III fused to protein A from *S.* *aureus* (soluble)	*B. occitanus tunetanus*	*E. coli*	Mice	Venom of *B. occitanus tunetanus*	10	Aah-G50	[22]
AaH1, AaH2, AaH3 fused to MBP (soluble)	*A. australis hector*	*E. coli*	Rabbits	Aah-G50 fraction	15	ND	[23]
Native AmmVIII	*A. mauretanicus*	Venom	Rabbits	Native AaH2	42	AahII	[24]
Ts1-Ts1 (IB)^a^	*T. serrulatus*	*E. coli*	Rabbits	Venom and native Ts1	15	ND	[25]
Css2 fused to 6His (IB)	*C. suffusus suffusus*	*E. coli*	Rabbits	Native CssII and Cn2/venoms from *C. noxius* and *C. s. suffusus*	12	*C. noxius*	[26]
Pg8 fused to 6His (IB)	*P. granulatus*	*E. coli*	Mice	Native Pg8/venom	12	ND	[27]
Cn2, Css2, Cl1 fused to thioredoxin (soluble)	*C. noxius, C. s. suffusus, C. l. limpidus.*	*E. coli*	Rabbits	Venom from *C. noxius, C. suffusus suffusus, C. limpidus limpidus.*	12	*C. noxius, C. suffusus suffusus, C. limpidus limpidus*	[28]

IB: inclusion bodies; LD_50_: median lethal dose, which is the dose required to kill half the mice of a tested population.

Therefore, the present work reports the heterologous expression of the neurotoxins Acra4 and SccTx to produce immunogens in rabbits. Moreover, we suggest that a consensus of the North African scorpion toxin, like the group of AaH2 related toxins, and others associated with the group of Acra4 from the Middle East, could be used as immunogen rather than the complete scorpion venoms, to generate neutralizing antibodies, and such polyvalent antibodies could be useful for improving scorpion antivenoms.

## Methods

### Venoms and toxins

Venoms used in this study were from different sources. *Androctonus crassicauda* venom was from Sanliurfa province of Turkey. *A. australis* and *A. mauritanicus, Leiurus quinquestriatus hebraeus,* and *Buthus occitanus* venoms were kindly donated by Eskisehir Osmangazi University, and by Dr. Lourival Possani and Dr. Alejandro Alagón from Instituto de Biotecnologia - UNAM. The freeze-dried venoms were dissolved in phosphate buffer (pH 7.0), centrifuged for 10 min at 17,600 *g*, and the supernatant was transferred to a clean tube. The neurotoxin AaH2 was purified as previously described [29] (Additional file 1).

### Animals

Mice of CD-1 strain (18-20 g) and Rabbits strain New Zealand (1.8-2 kg) were provided by facilities at the Instituto de Biotecnología, UNAM, México. Rabbits received regular veterinary supervision and were kept in suitable conditions and controlled environments. They received food and water *ad libitum*. Appropriate animal handling was always conducted to diminish suffering and discomfort, looking to maximize animal well-being during research following the Mexican legislation for the use of laboratory animals (Norma Official Mexicana, 1999, NOM-062-ZOO-1999). Moreover, animal management was in accordance with the Animal Care guidelines from the Bioethics Committee at the Instituto de Biotecnología, which administered and authorized the animal work (Ethical approval CB/IBt/Project #385).

### Neurotoxin sequence alignment

Sequences of α-neurotoxins from scorpion venoms were retrieved from the National Center for Biotechnology Information website (http://www.ncbi.nlm.nih.gov/pubmed) and the UniProt Protein knowledgebase (http://www.uniprot.org). Afterward, sequences were selected upon the lower median lethal dose and the higher medical relevance. The Jalview software performed a multiple sequences alignment of the α-neurotoxins and then specified a consensus amino acid sequence [30]. In positions with indeterminations, the amino acids chosen were the most abundant and those whose immunogenicity index was the best according to Kolaskar and Tongaonkar [31]. As a result, a consensus α-neurotoxin amino acid sequence was created and named SccTx (Table 1). 

### HisrAcra4 and HisrSccTx gene construction

First, the protein sequence of Acra4 was used to create a DNA sequence by reverse translation. Afterward, the sequence was evaluated to adjust the preferential codon usage of *E. coli* (http://www.kazusa.or.jp/codon). Four overlapping synthetic oligonucleotides (Additional file 2A) were proposed to build the Acra4 gene considering the DNA sequence. The oligonucleotides used were synthesized by the “Unidad de Síntesis y Secuenciación de DNA” at Instituto de Biotecnología. Additionally, restriction sites for *Bam*HI and *Pst*I were added in oligonucleotides forward (Acra4-Up1) and reverse (Acra4-Lw4), respectively. The coding sequence for protease Factor Xa (ATCGAGGGAAGG) was introduced after the *Bam*HI digestion site (Acra4-Up1, Additional file 2A) to obtain the mature toxin with no fusion protein. The Acra4 gene was assembled *in vitro* by “oligonucleotide overlapping extension” using polymerase chain reaction (PCR). Briefly, for the assembly of the complete gene, the four oligonucleotides were mixed in the same reaction Acra4-Up1 (0.4 pmol/µL), Acra4-Lw4 (0.4 pmol/µL), Acra4-Up2 (0.1 pmol/µL) Acra4-Lw3 (0.1 pmol/µL) and Vent-Pol (NEB). The PCR reaction was conducted at 61 ºC (annealing) during 30 cycles. The PCR product was run on 1 % agarose gels stained with GelRed® dye (Biotium) and revealed under ultraviolet (UV) light. Afterward, the amplificated DNA was purified from the agarose using the PCR Product Purification Kit (Roche®). 

Second, the primary structure of SccTx was obtained from seven primary structures of the most lethal scorpion neurotoxins from the Middle East and North Africa, including Acra4 (Table 1A). Therefore, four overlapping synthetic oligonucleotides (Additional file 2B) were designed to assemble the SccTx gene. Additionally, recognition sequences for *Bam*HI and *Pst*I were added to oligonucleotides forward (SccTx-Up1) and reverse (SccTx-Lw4), respectively. The coding sequence for protease TEV (Tobacco Etch Virus) (GAGAACCTGTACTTTCAAGGT) was introduced after the *Bam*HI digestion site to obtain the mature toxin with no fusion protein (SccTx-Up1, Additional file 2B). The SccTx gene was assembled *in vitro* by “oligonucleotide overlapping extension” using Polymerase Chain Reaction (PCR). Briefly, for the assembly of the complete gene, the 4 oligonucleotides were mixed in the same reaction. SccTx-Up1 (0.4 pmol/µL), SccTx-Lw2 (0.1 pmol/µL), SccTx-Up3 (0.1 pmol/µL), and SccTx-Lw4 (0.4 pmol/µL)) were mixed with Vent-Pol and amplified by PCR (60 ºC by 30 cycles). The PCR product was run on 1 % agarose gels stained with GelRed ® dye (Biotium) and revealed under ultraviolet (UV) light. 

The assembled genes (Acra4 or SccTx) were digested with *Bam*HI and *Pst*I enzymes. The digestions were run and extracted from agarose gel (1.2%) and subsequently ligated with the pQE30 vector, previously treated with the same enzymes. The recombinant plasmids (pQE30/Acra4 or pQE30/SccTx) were used to transform *E. coli* XL1-Blue cells by heat shock transformation and then plated in LB agar plus 100 µg/mL ampicillin. Ten colonies from each construction were evaluated by colony PCR (pQE-Fwd (GAGCGGATAACAATTATAA) and pQE-Rev (GGTCATTACTGGATCTAT)). Four colonies with an expected DNA amplification were cultivated with ampicillin, and their plasmids were purified and sequenced (Institute of Biotechnology, UNAM, Mexico).

### Bacterial strains, plasmids, and enzymes

*Escherichia coli* XL1-Blue was used for cloning and vector amplification. Meanwhile, *E. coli* Origami was utilized to produce HisrAcra4 or HisrSccTx. Plasmid pQE30 (Qiagen, CA, USA) was used for cloning and production of 6His-tagged recombinants HisrAcra4 or HisrSccTx. *Taq* polymerase, T4 DNA ligase, protease Factor Xa (FXa), and Restriction enzymes, were acquired from New England Biolabs (NEB, MA, USA). 

### Plasmid construction for expression

The designed genes were inserted in the expression vector pQE30, flanked by *Bam*HI and *Pst*I sites. The expressed genes contained several features such as a histidine-tag (6His) (to purify the produced peptides by affinity chromatography); a cleavage sequence (FXa) placed after the 6His and before the mature toxin of Acra4, and a cleavage sequence (TEV *Tobacco Etch Virus*) placed after the 6His and before the SccTx toxin (intended to obtain the full recombinant toxins). Cleavage sequences could be used if mature proteins (Acra4 or SccTx) were required (Additional file 3). The new recombinant plasmid sequences were confirmed (both ends). Each recombinant plasmid was used to transform competent *E. coli* Origami cells by heat shock and plated in LB agar, including ampicillin (100 µg/mL).

### Expression and purification of HisrAcra4 or HisrSccTx

Toxins from both plasmids, pQE30/Acra4 and pQE30/SccTx, were expressed in the *E. coli* strain Origami. The cells that produced HisrAcra4 were grown in MM medium [32], and the medium to produce HisrSccTx was Luria-Bertani LB. Once absorbance at 600 nm had reached 0.6 U, the cultures of pQE30/Acra4 and pQE30/SccTx were induced using 0.5 mM isopropyl-β-D-thiogalactopyranoside (IPTG) for 24 h at 16ºC, and 1 mM IPTG at 30ºC for 6 h respectively. Cells were centrifuged (20 min, 9,800 *g* in a JA-14 rotor), dissolved in washing buffer (0.05 M Tris-HCl, pH 8.0), and broken using a One-Shot Cell Disruptor (Constant Systems, Northants, United Kingdom). This material was centrifuged again (22,000 *g* for 20 min), and the supernatant was discarded.

The insoluble fraction was rinsed twice with washing buffer and centrifuged again for 20 min at 18,000 *g* in a JA-20 rotor. The supernatant was discarded, and the inclusion bodies contained in the insoluble fraction were dissolved with Tris-HCl 0.05 M buffer (pH 8.0) plus 6M guanidinium chloride (GndHCl) to extract the recombinant neurotoxins. The solutions were centrifuged for 20 min at 18,000 *g* (JA-20 rotor Beckman centrifuge) to eliminate insoluble materials. The supernatant that contains the recombinant protein was purified by Ni-NTA (Ni-nitrilotriacetic acid) affinity column chromatography, which was performed according to the manufacturer’s instructions (Qiagen, CA, USA), using denaturing conditions with buffer A (6 M GndHCl in a 0.05M Tris-base buffer, pH 8.0) and buffer B (6 M GndHCl in 0.05 M Tris-base buffer, containing 400 mM imidazole, pH 8.0). The recombinant protein was then purified by applying a second purification step under Reversed-Phase High Performance Liquid Chromatography (RP-HPLC) using an analytical C_18_ reversed-phase column (Vydac 214 TP 4.6 x 250 mm, Hesperia, CA, USA) with a linear gradient using solvent A (0.1% trifluoroacetic acid, TFA, in water) and solvent B (0.1% TFA in acetonitrile). The linear gradient was from 0 to 60 % of solvent B over 60 min at 1 mL/min flow rate, with UV detection at 230 nm. The GndHCl of buffer B was eliminated during this purification step. The HisrAcra4 and HisrSccTx were vacuum dried, and later allowed to fold under controlled conditions using 2M GndHCl in 0.05 M Tris-base buffer, pH 8.0, containing 8 mM reduced cysteine (Cys)/ 1 mM oxidized cystine (Cys-Cys) for 24 h at room temperature. Once the folding reaction occurred, it was purified again using the same RP-HPLC protocol.

### Molecular mass determination

The molecular mass of the recombinant proteins was determined by mass spectrometry. Briefly, 500 pmol of each protein was reconstituted in 5 μL of 50% acetonitrile with 1% acetic acid, and directly injected into a Thermo Scientific LCQ Fleet ion trap mass spectrometer (San Jose, CA, USA) with a Surveyor MS syringe pump delivery system. The eluate at 10 μL/min was split out to introduce only 5% of the sample into the nanospray source (0.5 μL/min). The spray voltage was set from 1.5 kV, and the capillary temperature was set to 150°C. The fragmentation source was operated at 25-35 V of collision energy, 35-45% (arbitrary units) of normalized collision energy, and the scan with wideband was activated. All spectra were obtained in the positive-ion mode. The data acquisition and the deconvolution of data were performed on the Xcalibur Windows NT PC data system.

### Circular dichroism

The secondary structure of the recombinant proteins HisrAcra4 and HisrSccTx was determined by circular dichroism (CD) spectroscopy. Spectra of the recombinant proteins were recorded within a wavelength ranging from 190 to 260 nm at room temperature in 1 mm-path quartz cells (spectropolarimeter Jasco J-710 (Jasco, Japan). Every 1 nm data were registered at 50 nm/min. Each protein was prepared to a concentration of 0.6 mg/mL in 60% trifluoroethanol, which increases secondary structure by sequestering water from the sampling solution. The CD values corresponded to the mean of three recordings. The spectra were deconvoluted employing the Beta Structure Selection webserver (BeStSel) http://bestsel.elte.hu/index.php [33].

### Animal immunization

Groups of rabbits were hyperimmunized subcutaneously with 6 mg of either HisrAcra4 or HisrSccTx to produce serum antibodies. The immunization protocols began by dispensing 0.01 mg of total protein mixed with Complete Freud´s Adjuvant (CFA); afterward, a growing dose of 0.2 mg mixed with aluminum hydroxide (AH) or incomplete Freud´s (IFA) was administered for one month. Then, the immunization process followed with 0.5 mg every week for 60 days. At the end of the immunization process, the serum from two rabbits was collected, and antibodies were extracted from plasma by acid precipitation (5% caprylic acid) according to standard procedures [34] and freeze-dried. A protein solution (50 mg/mL) from rabbit-derived immunoglobulins was kept at -20ºC until use.

### Electrophoretic analysis and western blotting of scorpion venoms and isolated toxins

Qualitative analysis of scorpion venoms and isolated toxins as native AahII, HisrAcra4, and HisrSccTx was performed using SDS-PAGE under reducing conditions [35]. The gels were stained using Coomassie Brilliant Blue R-250. The western blot assay required sample separation by SDS-PAGE and then proteins transference to a membrane (polyvinyl difluoride) 400 mA for one h (Owl semi-dry system). Afterward, the membrane was blocked with TBST (150 mM NaCl, 10 mM Tris, 0.5% Tween 20, pH 8.0) plus non-fat milk (5%) for 2 h at room temperature. Later, the blocking solution was discarded, and the membrane was washed with TBST (3 times) and then submerged in rabbit IgG anti-HisrAcra4 (1:500) or anti-HisrSccTx (1:500) for 1 h at room temperature and washed with TBST. A second incubation was done for 1 h at room temperature with a horseradish alkaline phosphatase-conjugated monoclonal anti-rabbit antibody (1:5000). Membranes were washed with TBST (3 times) and revealed by BCIP/NBT (Invitrogen) according to the manufacturer´s protocols.

### Enzyme-linked immunosorbent assay (ELISA)

Venoms and expressed toxins were solid-phase-adsorbed by treating wells of MaxiSorp plates (NUNC™, Thermo Scientific, Waltham, MA, USA) with 100 µL solution containing 5 µg/mL of venoms and each recombinant protein in 100 mM sodium carbonate buffer (pH 9.6). Plates were incubated at 4°C overnight; afterward, wells were aspirated and washed three times with 200 µL of washing buffer (50 mM Tris-HCl pH 8 + 150 mM NaCl + 0.05% Tween 20). Subsequently, wells were filled with 200 µL of blocking buffer (50 mM Tris-HCL pH 8.0 + 0.5% gelatin + 0.2% Tween 20). After 2 h of incubation at 37°C, wells were washed as before and added with 100 µL aliquots, with serially diluted rabbit IgGs anti-HisrAcra4 or anti-HisrSccTx in incubation buffer (50 mM Tris-HCl buffer at pH 8 + 500 mM NaCl + 0.1% gelatin + 0.2% Tween 20). Dilution was initiated at 1:30, and then incubated at 37°C for 1 h. Later, the wells were washed, and the rabbit IgGs bound to the well reacted with 100 µL of incubation buffer containing 0.1 mg/mL of anti-rabbit IgGs, coupled with horseradish peroxidase (Merck KGaA, Darmstadt, Germany), and incubated for one hour at 37°C. Afterward, wells were emptied, washed, and filled with ABTS solution (100 µL) (Roche, Basel, Switzerland) as the substrate for peroxidase. The reaction must develop a color that was arrested by adding SDS solution (20%, 25 µL). The absorbance was measured at 405 nm in a Microplate Reader (Tecan Sunrise IVD version, Tecan Trading AG, Switzerland). The data were analyzed by nonlinear regression using the sigmoidal dose-response equation (GraphPad Prism v. 6.0c, San Diego, CA, USA). Titers were estimated from the middle of the curve and corresponded to the IgGs dilution for half of the maximal binding. This was contemplated as half the maximal effective concentration (EC_50_).

### Biological activity

The neurotoxic activity of HisrAcra4 and HisrSccTx was determined by intracranial injections in mice according to Pedigo et al. [36]. Briefly, mice were divided into two groups with three mice in each group: normal saline and recombinant toxin (1 μg/mouse). Mice were manually restricted, and the sample was injected through the skull at a volume of 5 μL within a puncture point 2 mm lateral to bregma, using a syringe with a truncated 27-gauge needle in a fixed tube so that it penetrated the brain only 3 mm from the top of the skull. The mice were observed for 1 min after the administration.

### Protecting activity of immunoglobulins

Male mice (CD-1, 18-20 g body weight) were injected intravenously (IV) in the tail using IgGs obtained from rabbits. The experiments followed the guidelines of our Institute Committee of Animal Welfare, with a minimum quantity of animals to corroborate the experimentations. For neutralization experiments, 3LD_50_ of whole *Androctonus crassicauda*, *A. australis, A. mauritanicus, Leiurus quinquestriatus hebraeus*, and *Buthus occitanus* venoms, as well as the AaH2 toxin, was incubated 30 min at 37ºC with either anti-HisrAcra4 or anti-HisrSccTx antibodies and then injected by IV via [37]. The alive mice were counted after 48 h.

### Statistics

The Prism 6.0 software (GraphPad Inc., San Diego, CA) was utilized to analyze the results. For ELISA, slope variable nonlinear regression analyses were performed [log(inhibitor) vs. response (three parameters)]. For all statistical analyses, as well as for the determination of mean values, standard deviations, coefficients of variation, and 95% confidence intervals, Prism 6.0 software (GraphPad, CA) was also used. One-step ANOVA test, multiple comparisons test and Tukey’s test were used in the analysis of biological tests (significant p-value < 0.05).

## Results and discussion

### Amino acid sequences of Acra4 and consensus SccTx compared to known neurotoxins of scorpion venoms from North Africa and the Middle East

As mentioned, Acra 4 and the toxin AaH2 are two types of proteins that differ slightly in their primary structures and belong to two distinct structural and immunological groups of neurotoxins (Table 1). We selected seven α-neurotoxins, including Acra4, from the venom of scorpions from North Africa and the Middle East. Such neurotoxins contain 65 to 66 amino acid residues with LD_50_s ranging from 0.18 to 0.5 µg/20 g mouse, determined intracranially or subcutaneously (Table 1A and 1B). Such selected α-neurotoxins are structures that have been verified for their presence in their respective venoms and they have been tested for their biological activity and proved to be lethal to mammals. Unfortunately, several α-neurotoxins have been reported from Sanger or massive DNA sequencing, and most of them have been neither proved their existence in their venoms nor their biological activity. Acra4 has the lower amino acid sequence identity compared to six primary structures of neurotoxins of the family belonging to the AaH2 neurotoxin (< 60%), but it has a much better identity (> 60%) compared to other neurotoxin primary structures belonging to the AaH1 and AaH3 neurotoxins (Table 1B). Table 1 also shows the slightly different amino acid residues (underlined in Table 1A) between these two families of mammalian neurotoxins.

Furthermore, a consensus sequence, SccTx, that represents the AaH2 family of scorpion neurotoxins, was built based on a multiple sequence alignment using particular α-neurotoxins (Table 1A). The toxin has 67 amino acids; nevertheless, four residues were undetermined (Table 1A). Intended to fulfill each undetermined residue, a corresponding amino acid was selected upon the following conditions: frequency, similar chemical properties, or higher immunogenicity. Consequently, the undetermined residues of SccTx were complete with L1, K10, K27, and K66. Finally, the SccTx sequence contained 67 amino acid residues [12 positively charged, 7 negatively charged, and 8 cysteines (Table 1B)].

### Gene construction

First, the synthetic gene for the antigenic group coding for Acra4 was constructed. To the N-terminus of Acra4, sixteen extra amino acids (MRGSHHHHHHGSIEGR), including a His-Tag from the pQE-30 vector, the restriction sequence for *Bam*HI, and the proteolytic FXa site were included. Therefore, the HisrAcra4 has 81 residues (Additional file 3A). Second, the synthetic gene for the antigenic group coding for the SccTx was constructed. To the N-terminus of SccTx, nineteen extra amino acids (MRGSHHHHHHGSENLYFQG), including the His-Tag region (coming from pQE-30 plasmid), *Bam*HI restriction site, and the TEV protease site were added. Therefore, the HisrSccTx has 86 residues (Additional file 3B).

### Expression, purification, and folding of HisrAcra4 and HisrSccTx proteins

**Figure 1.**
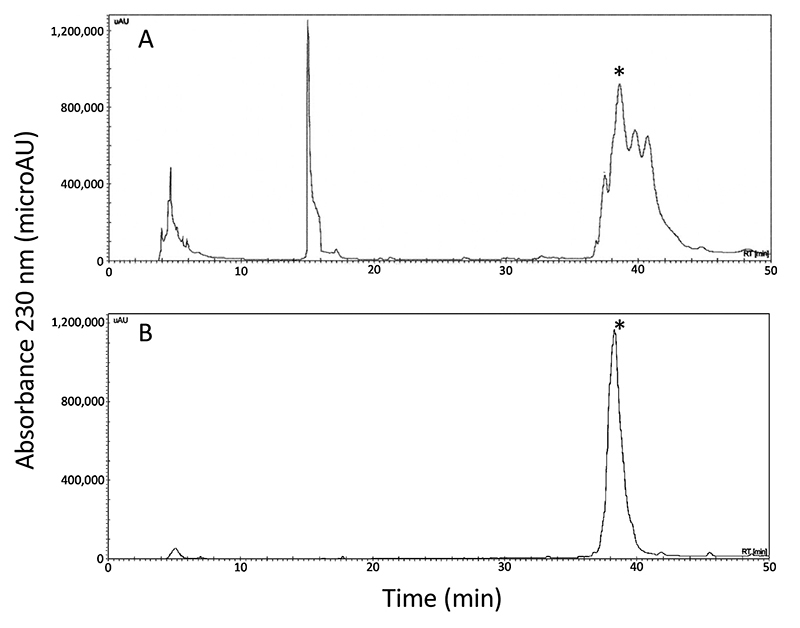
RP-HPLC separation of HisrAcra4 **(A)** by affinity chromatography and **(B)** after re-purification. **(A)** The recombinant protein HisrAcra4 was purified by affinity chromatography elution by applying RP-HPLC using an analytical C_18_ reversed-phase column (Vydac 214 TP 4.6 x 250 mm, Hesperia, CA, USA) with a linear gradient using solvent A (0.1% trifluoroacetic acid, TFA, in water) and solvent B (0.1% TFA in acetonitrile). The linear gradient was from 0 to 60 % of solvent B over 60 min at 1 mL/min flow rate, with UV detection at 230 nm. **(B)** The re-purification step used similar RP-HPLC conditions as in A). The asterisk shows the fraction which presented lethal activity.

**Figure 2.**
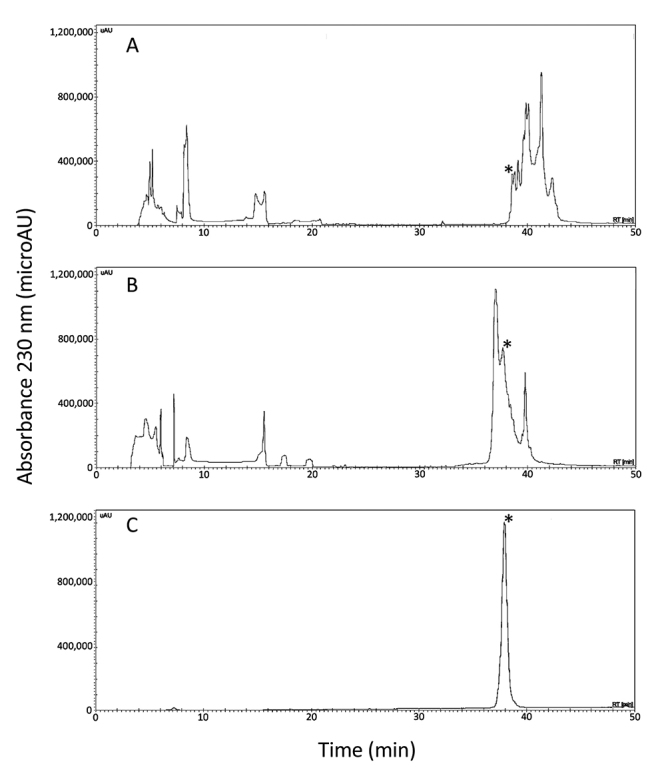
Chromatographic separation before and after protein *in vitro* folding of HisrSccTx **(A)** by affinity chromatography before refolding, **(B)** after purification by refolding and **(C)** after re-purification. **(A)** The recombinant protein HisrSccTx was purified by affinity chromatography elution by applying RP-HPLC using an analytical C_18_ reversed-phase column (Vydac 214 TP 4.6 x 250 mm, Hesperia, CA, USA) with a linear gradient using solvent A (0.1% trifluoroacetic acid, TFA, in water) and solvent B (0.1% TFA in acetonitrile). The linear gradient was from 0 to 60 % of solvent B over 60 min at 1 mL/min flow rate, with UV detection at 230 nm. **(B)** The purification of HisrSccTx after the refolding step used similar RP-HPLC conditions as in (A). **(C)** The re-purification step used similar RP-HPLC conditions as in A and B). The asterisk shows the fraction which presented lethal activity.

The synthetic genes encoding HisrAcra4 and HisrSccTx were built and cloned into the expression plasmid pQE30, which confers an N-terminal 6His-tag to expressed proteins, allowing the purification of the heterologous toxins by nickel affinity (NiNTA). Cleavage sites, FXa or TVE, were added among the 6His-tag and the mature HisrAcra4 or HisrSccTx, respectively, in the event of a possible undesirable biological outcome of the 6His-tag concerning the activity of each neurotoxin. In this situation, the tag could be removed from the respective protein. Heterologous expressions of either HisrAcra4 or HisrSccTx were performed in the *E. coli* Origami strain. HisrAcra4 or HisrSccTx were predominantly found in inclusion bodies and were dissolved using a chaotropic agent and then recovered by agarose nickel affinity. Western-blot assays allowed confirmation of heterologous expressions of HisrAcra4 or HisrSccTx in either inclusion bodies, or Ni-NTA fractions, using an anti-6His-tag antibody combined with alkaline phosphatase (data not shown). HisrAcra4 was purified directly in two steps by Reversed-Phase High Performance Liquid Chromatography (RP-HPLC) from after agarose nickel columns (Figure 1). An active form of HisAcra4 was observed in the first step of purification by RP-HPLC (Figure 1A). Therefore, a subsequent fractionation step was necessary to obtain the active form of HisrAcra4 pure (Figure 1B). On the other hand, HisrSccTx required an *in vitro* folding step to improve the yield of its active form (Figure 2B), and a third purification step to obtain the active form of HisrSccTx pure (Figure 2C). HisrAcra4 or HisrSccTx were subjected to mass spectrometry showing the experimental molecular masses of 8,947.6 and 9,989.1 Da in their oxidized form (Figures 1 and 2) that agree to the theoretical molecular masses of HisrAcra4 (8,947.2 Da) and HisrSccTx (9,989.1 Da), respectively (Additional files 4 and 5). Both proteins contain 8 cysteines forming four disulfide bonds. Therefore, the fractions identified from the RP-HPLC matched the molecular masses for HisrAcra4 or HisrSccTx. Since both HisrAcra4 and HisrSccTx were active, the folding problem was overwhelmed; therefore, removing the fusion tag containing the polyHis chain was unnecessary. The recombinant protein yield for HisrAcra4 and HisrSccTx was 2 and 1.5 mg/L, respectively. 

### Secondary structure of HisrAcra4 or HisrSccTx

**Figure 3.**
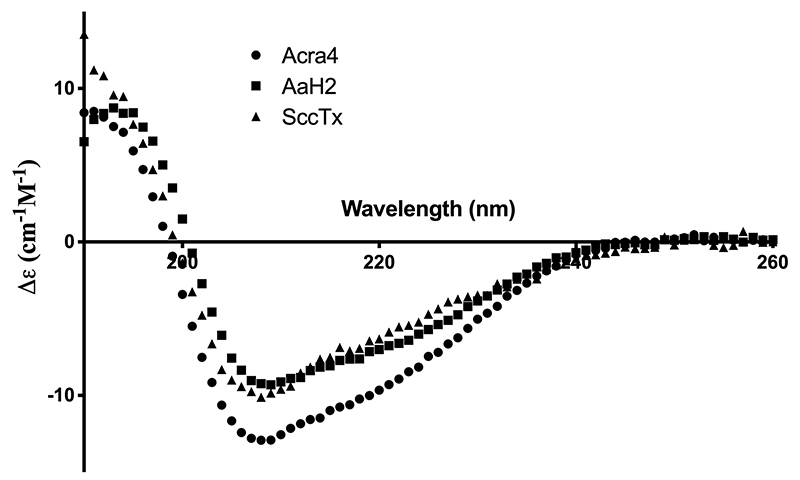
Circular dichroism of recombinant proteins HisrAcra4, HisrSccTx and native AaH2. The secondary structure fractions of the CD spectra according to Micsonai et al. [32] were: α-helix 0.36, 0.42, 0.34; β-sheet 0.21, 0.12, 0.10; and random coil 0.35, 0.25, 0.35 for HisrAcra4, HisrSccTx and native AaH2, respectively.

After protein expression and purification and proving that both HisrAcra4 and HisrSccTx were active, the secondary structure of the recombinant neurotoxins was analyzed by CD and compared to AaH2 (Figure 3). The three neurotoxins registered elevated absorption for α-helix secondary structures and β-strand contents agreeing with the CD deconvolution software [32]. So far, mostly all scorpion neurotoxins that affect Nav from the “old world” comprise a larger ratio of α-helix, described by negative ellipticities (208-222 nm) and a positive band (198 nm) [18, 38, 39]. Similar secondary structure fractions using aqueous trifluoroethanol (TFE) have been previously observed in Na^+^ channel scorpion toxins containing α/β structures [29, 38]. Here, HisrAcra4 or HisrSccTx showed similar patterns to the native neurotoxin AaH2. Therefore, the two recombinant proteins resemble canonical secondary structures of scorpion venom neurotoxins [38, 40]. 

### Rabbit immunization, titers, and antibody recognition

**Figure 4.**
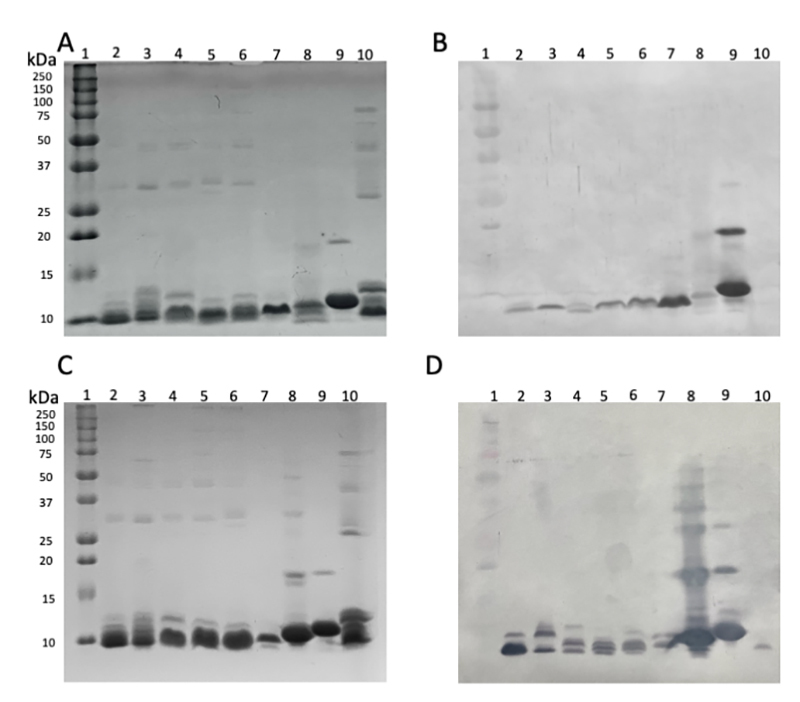
Proteins contained in venoms from different species of North Africa and the Middle East scorpion venoms were detected by SDS-PAGE and Western-blot. **(A)** SDS-PAGE 12.5%, **(B)** Western blot anti-HisrAcra4, **(C)** SDS-PAGE 12.5%, **(D)** Western blot anti-Scctx. Lanes: 1 - PM, 2 - *A. crassicauda,* 3 - *A. australis hector*, 4 - *A. mauritanicus,* 5 *- L. quinquestriatus hebraeus.* 6 - *B. occitanus,* 7 - native AaH2, 8 - HisrAcra4, 9 - HisrSccTx, 10 - *T. pachyurus.* **(A)** The SDS-Gel had 50 µg per well, and the Western-blot had 10 µg per line (first antibody: rabbit IgG anti-mixture of recombinants HisrSccTx; second antibody: rabbit IgG combined to alkaline phosphatase).

**Figure 5.**
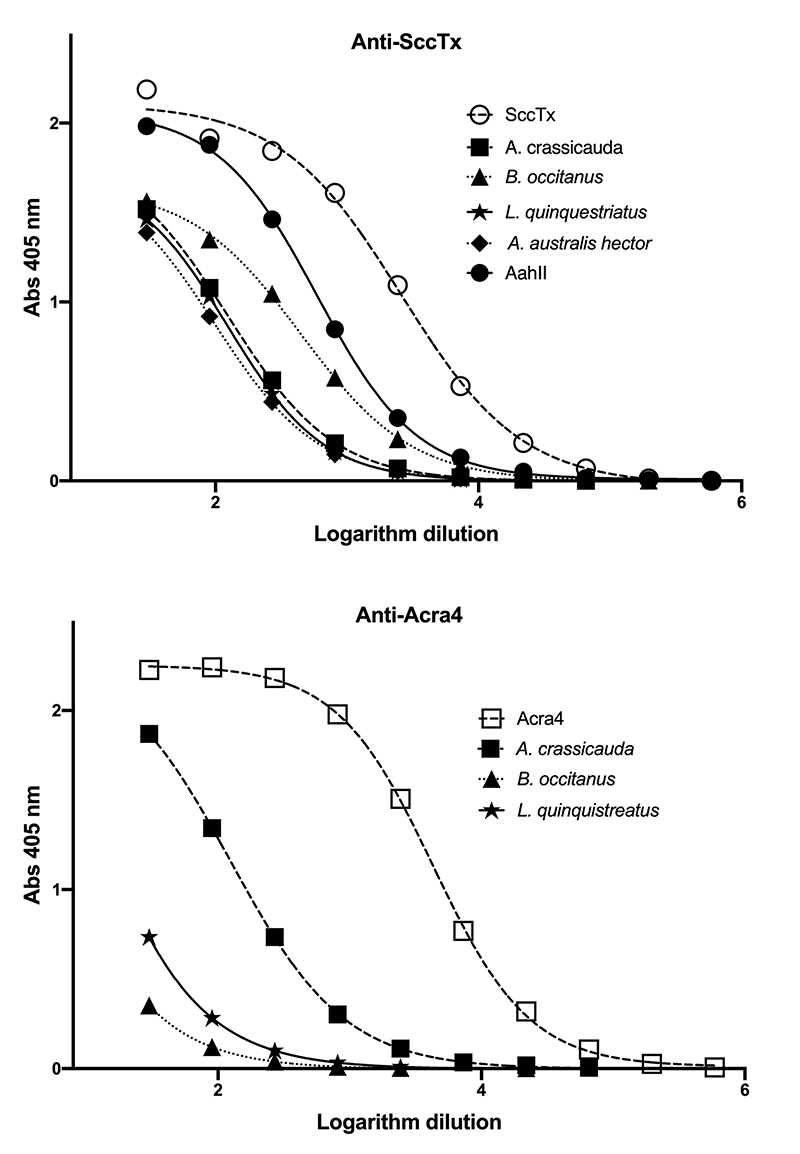
Antibody recognition of rabbit IgGs to venoms from North Africa and Middle East scorpion species. Rabbit hyper-immune sera raised against HisrAcra4 or HisrSccTx were titrated by ELISA using different venoms of North Africa and Middle East scorpion species. Points represent the mean ± SD of triplicate wells of the ELISA.

Groups of rabbits were immunized with 6 milligrams of either HisrAcra4 or HisrSccTx. After immunization (3 months), the rabbits were bled, and the serum capability to recognize each recombinant toxin and related scorpion venoms was tested. The protein content of scorpion venoms of species from North Africa and the Middle East was displayed by SDS-PAGE. Also, the western blot of the same venoms revealed the capability of the rabbit sera to recognize scorpion components and the recombinant toxins HisrAcra4 or HisrSccTx, respectively. The SDS-PAGE gel showed the expected apparent molecular mass of neurotoxins and enzymes in the venom of North Africa and Middle East scorpion species (Figures 4A and 4C). Also, Figures 4B and 4D illustrate how rabbit antibodies recognize venom proteins in those scorpions. Antibody recognition was detected for toxins in a narrow range, representing the native neurotoxins from scorpion venoms. Furthermore, Figure 5 shows the rabbit antibody titers (AT_50_) against the heterologous neurotoxins and the whole venom of scorpion species from North Africa and the Middle East. The AT_50_ values are here expressed as the dilution of serum giving half-maximal binding, and they represent the dilution of plasma that achieves 50% of maximal binding. The recombinant neurotoxins were the strongest identified, as expected. HisrAcra4 had the better AT_50_ compared to HisrSccTx. Concerning the recognition of the rabbit antibodies against the scorpion venoms*,* the venom from *A. crassicauda* was the best documented, with AT_50_ values of 124 for the anti-HisrAcra4 antibodies, and *B. occitanus* was the best recognized, with AT_50_ values of 446 for anti-HisrSccTx (Table 3). Although antibody detection was unmistakable, the AT_50_ values for anti-HisrAcra4 were very low for the venoms from *L. q. hebraeus* and *B. occitanus* with AT_50_ values of 8.9 and 1.9, respectively. The antibodies against HisrSccTx had AT_50_ values of 98, 124, and 123 for the venoms from *A. australis hector, A. crassicauda,* and *L. q. hebraeus*, respectively, and the most recognized venom was *B. occitanus* and the AaH2 neurotoxin with AT_50_ values of 446 and 590, respectively (Table 3).

**Table 3.** Antibodies titers from rabbits *versus* the recombinant HisrAcra4, HisrSccTx and scorpion venoms

	Anti-HisrAcra4		Anti-HisrSccTx	
Protein	AT_50_	CI	AT_50_	CI
HisrAcra4	4,290	3,646-5,048	ND	ND
HisrSccTx	ND	ND	2,578	2,147-3,069
*A. australis hector*	ND	ND	98	77-116
*A. crassicauda*	124	121-127	124	102-145
*L. q. hebraeus*	8.9	7.2-11.1	123	119-127
*B. occitanus*	1.9	0.5-6.9	446	408-485
AaH2	ND	ND	590	561-619

CI: confidence intervals (95%); ND: values not determined; n = 3.

### Venom neutralization

The efficiency of antivenoms is based on their ability to neutralize the lethal effects of venoms. Therefore, anti-HisrAcra4 or anti-HisrSccTx rabbit IgGs were challenged with three times the amount of their median lethal dose (LD_50_) of *Androctonus crassicauda*, *A. australis, A. mauritanicus, Leiurus quinquestriatus hebraeus*, and *Buthus occitanus* venoms as well as the native AaH2 toxin [37]. The anti-HisrAcra4 antibodies could not neutralize any scorpion venoms tested (Table 4). Nevertheless, ten milligrams of anti-HisrSccTx IgGs were enough to neutralize such venoms, except the ones from *A. crassicauda* and *A. mauritanicus*. However, a blend of anti-HisrAcra4 and anti-HisrSccTx IgGs neutralized the lethality of *A. crassicauda* and *A. mauritanicus* (Table 4). These results are interesting; that is, all scorpion venoms tested here contain the two types of mammalian neurotoxins, Acra4, AaH1, and AaH3, which represent one group, and the neurotoxins such AaH2, AmmV, and AmmVIII, which represent another group (see Table 1A and 1B). However, the antibodies anti-HisrAcra4 could not neutralize the scorpion venom, which it may be caused because of the presence of α-neurotoxins in the AaH2 group. Usually, the number of α-neurotoxins of the AaH2 group in these types of scorpion venoms is more abundant than that of the AaH1/AaH3 group [12, 13, 41, 42]. Moreover, the venoms of *A. crassicauda* and *A. mauritanicus* were not neutralized by the anti-HisrSccTx IgGs, which could be because such antibodies failed to neutralize α-neurotoxins of the AaH1/AaH3 group. Still, the blend of anti-HisrAcra4 and anti-HisrSccTx IgGs successfully neutralized the venoms of *A. crassicauda* and *A. mauritanicus.* These results shed light on the importance of both groups of neurotoxins, the AaH2 and the AaH1/AaH3 group in scorpion venoms from the Middle East and North Africa. The commercial antivenoms have to be able to neutralize both types of α-neurotoxins.

**Table 4.** Venom and toxin neutralization using antibodies again HisrAcra4 and HisrSccTx.

Venom or Toxin	LD_50_ µg/mice	LD_50_ (iv)^a^	3LD_50_ (µg)	Antibody used	Antibody (mg)	Mice/Alive	**µg V/** **mg AV**
*A. australis hector*	4.5	3	13.5	None (Control)	0	3/0	1.35
	4.5	3	13.5	Anti-SccTx	10	3/3	
*A. crassicauda*	6.4	3	19.2	None (control)	0	3/0	0.96
	6.4	3	19.2	Anti-Acra4	10	3/0	
	6.4	3	19.2	Anti-SccTx	10	3/0	
	6.4	3	19.2	Anti-SccTx + Anti-Acra4	10 each (20 total)	3/3	
*A. mauritanicus*	4.7	3	14.1	None (control)	0	3/0	0.70
	4.7	3	14.1	Anti-Acra4	10	3/0	
	4.7	3	14.1	Anti-SccTx	10	3/0	
	4.7	3	14.1	Anti-SccTx + Anti-Acra4	10 each (20 total)	3/3	
*L. quinquestriatus hebraeus*	4.2	3	12.6	None (control)	0	3/0	1.26
	4.2	3	12.6	Anti-SccTx	10	3/3	
*B. occitanus*	8.5	3	25.5	None (control)	0	3/0	2.46
	8.5	3	25.5	Anti-SccTx	10	3/3	
AaH2	0.18*	5.5	1.0	No (control)	0	3/0	0.1
	0.18*	5.5	1.0	Anti-SccTx	10	3/3	

^a^ 3LD_50_ were intravenously injected into mice, and it means three times the LD_50_. *The LD_50_, median lethal dose, is subcutaneous according to Alami et al. [12]; 3LD_50_ were used to calculate the µg venom/mg AV.

## Conclusions

The current research demonstrates the proof of concept of the use of recombinant Acra4 toxin and consensus SccTx toxin to produce antibodies against some antigenic groups of neurotoxins and venoms from North African and the Middle Eastern scorpions. Although there are communications on the use of these two types of neurotoxins (produced recombinantly) for antivenom production against specific scorpion venoms species, none of such reports have pointed out the importance of using separately these two types of proteins as immunogens for raising antibodies to neutralize larger scorpion venom species from these geographical regions. Moreover, most of such reports regrettably have not indicated the process of neurotoxin folding leaving a gap in the process of obtaining a well-folded protein, especially when they contain several cysteines to form a considerable number of structurally different isoforms because of the large disulfide connectives that they could form. The neutralization of North African and the Middle Eastern scorpion venoms using antibodies against the recombinant Acra4 toxin and the consensus SccTx toxins sum to the communications for applying recombinant antigens for antivenom production [32, 39, 43, 44]. Currently, scorpion antivenoms used for therapeutic pursuits are produced in animals as polyclonal antibodies, mainly using complete scorpion venom as immunogens. However, new strategies are being reviewed [32], such as using heterologous proteins from venoms to produce monoclonal or polyclonal antibodies [43, 44].

## Supplementary material

The following online material is available for this article:

Additional file 1. Chromatographic separation by RP-HPLC of the venom of *A. australis hector*. Molecular masses of different fractions obtained by mass spectrometry are shown. The fraction corresponding to the AaH2 toxin is shown in gray.

Additional file 2. The first forward and the last reverse oligonucleotides were used for HisrAcra4 and HisrSccTx assembly. The structural elements added to the sequence of the recombinant toxin are shown (the *Bam*HI and *Pst*I sites, as well as the stop codons, are shown).

Additional file 3. Representation of the gene construction for the heterologous expression of Acra4 and SccTx. The primary structure of **(A)** Acra4 and **(B)** SccTx in bold. The 6His-coding sequence is part of the pQE30 vector and is located upstream of the *Bam*HI/*Pst*I-cloned gene; so, the recombinant protein gets 6His-tagged at the amino terminus (cursive). Downstream of the *Bam*HI site, the sequence coding for either **(A)** FXa or **(B)** TEV recognition site are introduced (IEGR or ENLYFQG is underlined) right before the mature toxin’s sequence, respectively. Two stop codons (asterisks) are included at the end of the sequence coding for the mature toxin, upstream of the *Pst*I cloning site.

Additional file 4. Mass spectrometry analysis of HisrAcra4.

Additional file 5. Mass spectrometry analysis of HisrSccTx.
