# Supplementary material for: Recombinant expression and antigenicity of two peptide families of neurotoxins from Androctonus sp
Source: J Venom Anim Toxins Incl Trop Dis. 2022 Dec 19;28:e20220026. doi: 10.1590/1678-9199-JVATITD-2022-0026 (PMC9769139; doi:10.1590/1678-9199-JVATITD-2022-0026)
Supplement: Additional file 1. [file 1678-9199-jvatitd-28-e20220026-s1.pdf]

J Venom Anim Toxins incl Trop Dis.

https://doi.org/10.1590/1678-9199-JVATITD-2022-0026

Supplementary Material to "Recombinant expression and antigenicity of two peptide families of neurotoxins from *Androctonus* sp.*"*

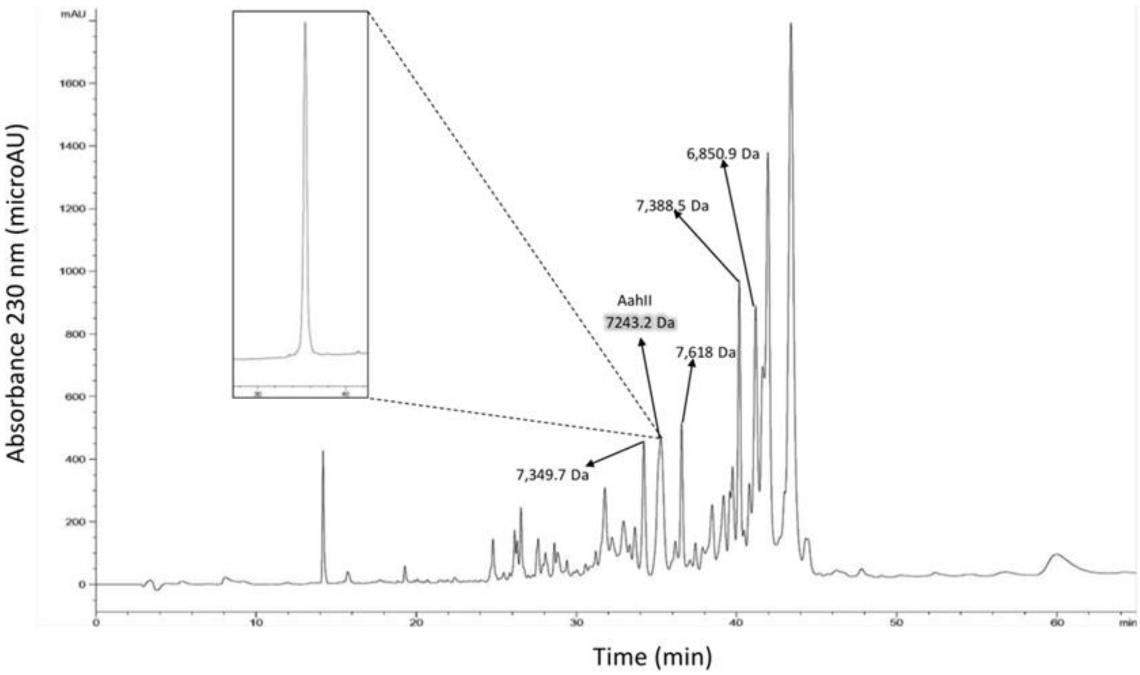

Additional file 1. Chromatographic separation by RP-HPLC of the venom of *A. australis hector*. Molecular masses of different fractions obtained by mass spectrometry are shown. The fraction corresponding to the AaH2 toxin is shown in gray.
