# Supplementary material for: Recombinant expression and antigenicity of two peptide families of neurotoxins from Androctonus sp
Source: J Venom Anim Toxins Incl Trop Dis. 2022 Dec 19;28:e20220026. doi: 10.1590/1678-9199-JVATITD-2022-0026 (PMC9769139; doi:10.1590/1678-9199-JVATITD-2022-0026)
Supplement: Additional file 2. [file 1678-9199-jvatitd-28-e20220026-s2.pdf]

J Venom Anim Toxins incl Trop Dis.
https://doi.org/10.1590/1678-9199-JVATITD-2022-0026

Supplementary Material to "Recombinant expression and antigenicity of two peptide families of neurotoxins from *Androctonus* sp."

Additional file 2. The first forward and the last reverse oligonucleotides were used for HisrAcra4 and HisrSccTx assembly. The structural elements added to the sequence of the recombinant toxin are shown (the *Bam*HI and *Pst*I sites, as well as the stop codons, are shown).

(A) DNA sequence, four overlapping synthetic oligonucleotides used for HisrAcra4 assembly.

Description	Oligonucleotides (5' →3')
Acra4 -Up1<br>Length 77 nt	GAG *GGA TCC* ATC GAG GGA CGC GTG CGT GAC GGC TAC ATC GTG GAT GAC AAA AAT TGT GTG TAT CAT TGC ATT CCT CC
Acra4 -Lw2<br>Length 70 nt	CTA CAA CTG CCG CTC TTA CCG CCG TTT TTC TTG CAC AGA CCA TCA CAC GGA GGA ATG CAA TGA TAC ACA C
Acra4 -Up3<br>Length 68 nt	GTA AGA GCG GCA GTT GTA GCT TTC TGG TTC CGA GTG GTT TAG CGT GCT GGT GTA AAG CCC TGC CTG AC
Acra4 -Lw4<br>Length 76 nt	CTC CTG CAG **CTA TTA** GCG CTT GTG GCA TTT GTA GCT AGG GTC CTT GAT CGG CAC ATT GTC AGG CAG GGC TTT A CA C

Site BamHI: *GGA TCC*; Site FXA: ATC GAG GGA CGC; Site STOPs: **CTA TTA**; Site PstI: CTG CAG.

(B) DNA sequence, four overlapping synthetic oligonucleotides used for HisrSccTx assembly.

Description	Oligonucleotides (5' →3')
SccTx-Up1<br>Length 78 nt	GAG A*GG ATC C*GA GAA CCT GTA CTT TCA AGG TCT GAA AGA CGG CTA TAT TGT GGA TGA CAA GAA CTG CAC CTA CTT CTG
SccTx-Lw2<br>Length 69 nt	CTT TCG CCC TTC AGT TTC TTA CAC TCT TCG TTG CAA TAC GCA TTG CGA CCA CAG AAG TAG GTG CAG TTC
SccTx-Up3<br>Length 80 nt	GAA ACT GAA GGG CGA AAG CGG TTA CTG CCA GTG GGC AAG TCC GTA TGG CAA TGC CTG TTA CTG CTA TAA ACT GCC TGA TC
SccTx-Lw4<br>Length 77 nt	TCT CCT GCA G**CT ATT A**GC GTT TGT TAC AAC GGT AGC CCG GAC CCT TCG TGC GAA CAT GAT CAG GCA GTT TAT AGC AG

Site BamHI: *GGA TCC*; Site TEV: GAG AAC CTG TAC TTT CAA GGT; Site STOPs: **CTA TTA**; Site PstI: CTG CAG.

Genetic construction was used for the expression of the recombinant HisrAcra4 and HisrSccTx. The 6His-coding sequence is part of the pQE30 vector and is located upstream of the *Bam*HI/*Pst*I-cloned gene, so the recombinant protein gets 6His-tagged at the amino terminus. Downstream of the *Bam*HI site, the sequence coding for either the FXa or TEV recognition site is introduced (IEGR or TEV in bold) before the mature toxin's sequence. Two stop codons are included at the end of the sequence coding for the mature toxin, upstream of the *Pst*I cloning site.
