# Supplementary material for: Recombinant expression and antigenicity of two peptide families of neurotoxins from Androctonus sp
Source: J Venom Anim Toxins Incl Trop Dis. 2022 Dec 19;28:e20220026. doi: 10.1590/1678-9199-JVATITD-2022-0026 (PMC9769139; doi:10.1590/1678-9199-JVATITD-2022-0026)
Supplement: Additional file 3. [file 1678-9199-jvatitd-28-e20220026-s3.pdf]

J Venom Anim Toxins incl Trop Dis.
https://doi.org/10.1590/1678-9199-JVATITD-2022-0026

Supplementary Material to “Recombinant expression and antigenicity of two peptide families of neurotoxins from *Androctonus* sp.”

A

***Bam*H1**

-PT5-
*MRGSHHHHHHGS*IEGR**VRDGYIVDDKNCVYHCIPPCDGLCKKNGGKSGSCSFLVPSGLACWCKALPDNVPIKDPSYKCHK**
R**LQ

***Pst*1**

B

***Bam*H1**

-PT5-
*MRGSHHHHHHGS*ENLYFQG**LKDGYIVDDKNCTYFCGRNAYCNEECKKLKGESGYCQWASPYGNACYCYKLPDHVRTKG**
PGYRCNKR **LQ

***Pst*1**

Additional file 3. Representation of the gene construction for the heterologous expression of Acra4 and SccTx. The primary structure of **(A)** Acra4 and **(B)** SccTx in bold. The 6His-coding sequence is part of the pQE30 vector and is located upstream of the *Bam*HI/*Pst*I-cloned gene; so, the recombinant protein gets 6His-tagged at the amino terminus (cursive). Downstream of the *Bam*HI site, the sequence coding for either **(A)** FXa or **(B)** TEV recognition site are introduced (IEGR or ENLYFQG is underlined) right before the mature toxin's sequence, respectively. Two stop codons (asterisks) are included at the end of the sequence coding for the mature toxin, upstream of the *Pst*I cloning site.
